# Rationale and design of the multicenter randomized trial investigating the effects of levosimendan pretreatment in patients with low ejection fraction (≤40 %) undergoing CABG with cardiopulmonary bypass (LICORN study)

**DOI:** 10.1186/s13019-016-0530-z

**Published:** 2016-08-05

**Authors:** Thibaut Caruba, Delphine Hourton, Brigitte Sabatier, Dominique Rousseau, Annick Tibi, Cécile Hoffart-Jourdain, Akim Souag, Nelly Freitas, Mounia Yjjou, Carla Almeida, Nathalie Gomes, Pascaline Aucouturier, Juliette Djadi-Prat, Philippe Menasché, Gilles Chatellier, Bernard Cholley

**Affiliations:** 1 Department of Pharmacy, Hôpital Européen Georges Pompidou, AP-HP, Paris, France; 2 Clinical Trial Unit and INSERM CIC-141, Hôpital Européen Georges Pompidou, AP-HP, Paris, France; 3 Agence Générale des Equipements et des Produits de Santé (AGEPS), AP-HP, Paris, France; 4 Department of Anaesthesiology and Intensive Care, Hôpital Européen Georges Pompidou, AP-HP, 20 rue Leblanc, 75015 Paris, France; 5 Department of Cardiovascular Surgery, Hôpital Européen Georges Pompidou, AP-HP, Paris, France; 6 Université Paris Descartes, Sorbonne Paris Cité, Paris, France; 7 INSERM Centre de Recherche des Cordeliers UMR S 872 eq 22 Université Paris Descartes, Paris, France; 8 Département de la Recherche Clinique et du Développement (DRCD), Hôpital Saint-Louis, (AP-HP), Paris, France

**Keywords:** Coronary artery bypass graft, Low cardiac output syndrome, High risk surgical patient, Levosimendan, Cardiac surgery, Perioperative management

## Abstract

**Background:**

Patients with a left ventricular ejection fraction (LVEF) of less than 40 % are at high risk of developing postoperative low cardiac output syndrome (LCOS). Despite actual treatments (inotropic agents and/or mechanical assist devices), the mortality rate of such patients remains very high (13 to 24 %). The LICORN trial aims at assessing the efficacy of a preoperative infusion of levosimendan in reducing postoperative LCOS in patients with poor LVEF undergoing coronary artery bypass grafting (CABG).

**Methods/Design:**

LICORN study is a multicenter, randomized double-blind, placebo-controlled trial in parallel groups. 340 patients with LVEF ≤40 %, undergoing CABG will be recruited from 13 French hospitals. The study drug will be started after anaesthesia induction and infused over 24 h (0.1 μg/kg/min).

The primary outcome (postoperative LCOS) is evaluated using a composite criterion composed of: 1) need for inotropic agents beyond 24 h following discontinuation of the study drug; 2) need for post-operative mechanical assist devices or failure to wean from these techniques when inserted pre-operatively; 3) need for renal replacement therapy. Secondary outcomes include: 1) mortality at Day 28 and Day 180; 2) each item of the composite criterion of the primary outcome; 3) the number of “ventilator-free” days and “out of intensive care unit” days at Day 28.

**Discussion:**

The usefulness of levosimendan in the perioperative period has not yet been documented with a high level of evidence. The LICORN study is the first randomized controlled trial evaluating the clinical value of preoperative levosimendan in high risk cardiac surgical patients undergoing CABG.

**Trial registration number:**

NCT02184819 (ClinicalTrials.gov).

## Background

Patients with an ejection fraction of less than 40 % are at high risk of developing postoperative low cardiac output syndrome (LCOS) following cardiac surgery. The patients who develop this condition exhibit a very high mortality rate, ranging between 13 to 24 %, whereas those who are not affected by this complication have a mortality of 2 % only [1]. The LCOS is also associated with more frequent complications including pulmonary impairment, myocardial infarction, stroke, renal failure, and need for reoperation [2, 3]. Preventing LCOS is therefore a priority for anaesthesiologists working with cardiac surgical patients. The incidence of LCOS following coronary artery bypass grafting (CABG) surgery varies from 3 to 14 % [1], but the presence of a preoperative left ventricular dysfunction (i.e.: 20 % < left ventricular ejection fraction (LVEF) < 40 %) doubles the risk [1].

The therapeutic management of LCOS usually involves inotropic agents (catecholamines, phosphodiesterase inhibitors) as first-line treatment. Catecholamines may worsen myocardial oxygen imbalance and have been associated with an increase in morbidity and mortality after cardiac surgery [4]. Phosphodiesterase inhibitors such as milrinone are also associated with deleterious side-effects, such as hypotension and arrhythmia, and increased mortality in heart failure patients [4, 5]. New drugs that could help for the management of LCOS with a better tolerance profile are therefore highly desirable, in particular in view of the changing demographic profile of cardiac surgical patients whose severity at admission has steadily increased over the past years.

Levosimendan acts as an inotrope and a vasodilator via two different pathways. First, it induces a calcium-dependent binding to troponin C resulting in a “sensitization” of the myofilaments to calcium and a positive inotropic effect, without impairing myocardial oxygen balance. Second, it opens smooth muscle ATP-dependent potassium (K_ATP_) channels, thereby producing a vasodilatation. In addition, levosimendan also opens the mitochondrial K_ATP_ channels, an action that has been linked to a cardioprotective effect [6].

Several studies have suggested that levosimendan could be helpful to prevent LCOS and reduce morbidity and mortality after cardiac surgery [7–10]. Two recent meta-analyses on the perioperative use of levosimendan reinforce the idea of a potential benefit on outcomes, especially in patients with poor preoperative left ventricular ejection fraction [11, 12]. They also conclude that a large prospective randomized trial should be performed to confirm this hypothesis. The purpose of the “Levosimendan in coronary artery revascularization” (LICORN) trial is to verify the ability of levosimendan to prevent LCOS after CABG surgery in patients with poor left ventricular function.

## Methods/design

### Study population

We target the recruitment of patients aged over 18, scheduled for CABG surgery with cardiopulmonary bypass, who have a LVEF equal to, or less than 40 %, regardless of the method of assessment. The CABG may be combined (or not) with the repair of a valve or of another cardiac structure. The patients can be enrolled even if they receive preoperative inotropes, or if the surgeon decides to insert an intra-aortic balloon pump (IABP) prior to cardiopulmonary bypass, or if they have another left ventricular assist device in place. The exclusion criteria are listed in Table 1.

**Table 1:** Exclusion criteria

Preoperative renal failure (creatinine clearance < 30 ml/min)
Liver failure (prothrombine time < 50 %, in the absence of vitamin K antagonist
Cardiac surgery without CABG
Pregnancy
Emergency surgery, defined as surgery within the 24 h of the operative indication
Known allergy to levosimendan
Severe hypotension prior to surgery (mean arterial pressure < 60 mmHg)
Severe tachycardia prior to surgery (heart rate > 120 bpm)
Prior history of torsade de pointe
Dynamic obstruction of the left ventricular outflow tract
Lack of signed informed consent
Lack of affiliation to social security
Patient already involved in another trial

### Study design

Thirteen cardiac surgical centers, including 12 University hospitals and 1 private hospital, have been invited to participate in the study. The study is a randomized, double-blind, two-arm parallel-group, placebo-controlled phase III study. The chronology of the protocol is depicted in the Figure. Patients will be followed until postoperative day 180.

### Study drug administration

The study drugs are stored at 2–8 °C until reconstitution using 500 ml of 5 % dextrose solution, immediately prior to patient administration. Physical and chemical stability of the preparation at room temperature is demonstrated up to 24 h following reconstitution [13]. The placebo is made of riboflavin in order to reproduce the yellow colour of levosimendan. No bolus is administered. A continuous infusion of 0.1 μg/kg/min is started just after anaesthesia induction and is administered over the ensuing 24 h in the absence of serious adverse reaction. Tables are provided to clinicians to determine the flow rate of the drug solution according to patient’s weight. The study drug administration is discontinued in case of serious adverse events such as: 1) anaphylactic reaction, 2) refractory hypotension defined as a mean arterial pressure (MAP) < 60 mmHg despite optimal therapeutic response comprising fluids and vasoconstrictors at the discretion of the clinician, 3) intractable arrhythmia.

### Randomization and blinding procedures

The allocation of treatments will be performed by minimization (deterministic dynamic allocation) [14]. The procedure will take into account the center, the surgery performed (CABG alone, or combined surgery), LVEF (<30 %, or between 30 and 40 %), the redo nature of the surgery, the existence of a treatment by preoperative inotropic agents or IABP, and the existence of preoperative beta-blocker therapy. It will balance the distribution of prognostic factors between the two groups and within each center. We will introduce a random component to reduce the predictability of the allocation.

We chose to use a minimization procedure because the stratified randomization is not effective in trials with small sample size and large number of strata. This is the case in the present study since the 3 factors that must be controlled (type of surgery, preoperative treatment, and LVEF range) would result in eight strata at all 13 centers.

The randomization algorithm will be established by an independent statistician. This algorithm will then be installed on a website with secure access. At randomization, the clinician will obtain the blinded patient allocation number after having provided the patient characteristics required by the algorithm. The result of the randomization will be sent to the pharmacy at the center involved.

### Outcome measures

The primary aim of the trial is to demonstrate that levosimendan administration at the time of anaesthesia induction in patients undergoing CABG (alone, or combined with other cardiac repair) with poor LV function (LVEF ≤ 40 %) can reduce the incidence and severity of postoperative LCOS. Our primary endpoint is a composite of three elements that reflect this syndrome:

1. The need for inotropic agents beyond 48 h after the initiation of the study treatment infusion;
2. The need for post-operative circulatory mechanical assist devices (intra-aortic balloon pump, extra-corporeal life support, left ventricular assist device such as the Impella® pump) or failure to wean from these techniques (at 96 h following initiation of the study treatment) if they were inserted pre-operatively;
3. The need for renal replacement therapy (RRT) at any time during Intensive care unit stay (ICU).

The secondary goals are: 1) to compare mortality among placebo and levosimendan patients, and 2) to evaluate the effects of levosimendan treatment on ICU and hospital length of stay, as well as its impact on the duration of ventilation, and on each component of the primary composite endpoint separately. Thus, the secondary endpoints include:

1. Mortality at Day 28 and Day 180 (safety issue)
2. Individual components of the primary endpoint mentioned above
3. Number of days with mechanical circulatory assist devices
4. Number of days with renal replacement therapy and number of RRT kits that are used for each patient.
5. Number of ventilator-free days and out-of-ICU days at Day 28.

### Data collection

**Fig. 1:**
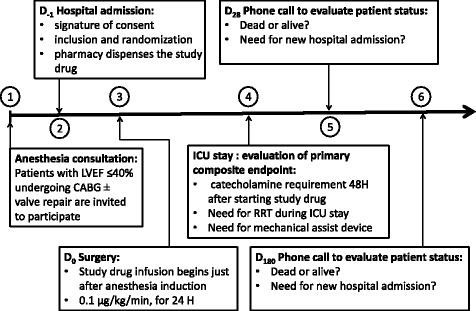
Schematic representation of the different steps of the LICORN protocol. LVEF: left ventricular ejection fraction; CABG: coronary artery bypass grafting; RRT: renal replacement therapy; ICU: intensive care unit

After enrolment into the study, patients are assigned a unique subject number associated with their initials. Data are collected by investigators with the help of clinical research assistants and recorded on paper case report forms (CRF). A CRF must be completed for each participant. The data entered into each CRF will be assessed by sponsor representatives, according to good clinical practices. In addition to the patient’s medical file, the investigator agrees to complete the documents related to treatment management provided by the sponsor and the CRF at each step of the protocol (Fig. 1). All corrections and alterations of data on the CRF must be made by the investigator him/herself and highlighted according to predefined instructions. The data management will be carried out by the Clinical Research Unit of Hôpital Européen Georges Pompidou. A list of variables of interest will be developed with an Operational Data Model format by the study primary investigator and the study statistician. This list will allow the implementation of the case report form and of the database. A data-management plan, developed jointly by the study coordinator, the data-manager, and the statistician will be implemented. After correcting for potential errors detected by this plan, a data review meeting will be held to validate the entire database with the study coordinator. The database will then be frozen prior to statistical analysis. The data will be analysed using SAS software (SAS Institute Inc., Cary, NC).

### Statistics

#### Number of subjects required

The number of subjects required is 340 patients (170 per group). This sample size has been obtained using the Nquery Advisor statistical software according to the following assumptions:

1. Incidence of the composite endpoint in the control group 65 % (proportion estimated from our personal data);
2. Incidence of the composite endpoint in the levosimendan group 50 %. According to Levin et al. [15], postoperative administration of levosimendan in patients having a LCOS has reduced by 28 % the use of an additional inotropic drug and by 12 % the use of a mechanical assist device, compared to dobutamine.
3. Alpha risk of 5 % and power of 80 %.

#### Statistical analysis

The results will be presented in accordance with the CONSORT 2010 recommendations. Estimates of the primary and secondary endpoints incidence will be provided with their 95 % confidence interval.

The primary analysis will be done on an intention to treat basis, in all patients included in the trial and having undergone randomization.

Incidence of the unadjusted composite endpoint will be compared between the two groups using a Chi-square test. A multiple logistic regression model will compare the effects adjusted for LCOS prognostic factors: type of surgery performed (CABG alone, or combined surgery), LVEF (<30 %, or between 30 and 40 %), redo nature of the surgery, preoperative treatment with inotropic drugs or mechanical assist device and presence of preoperative beta-blocker therapy.

Three subgroups analyses are planned. They will assess whether the levosimendan effect varies according to:

- Type of surgical procedure performed (CABG alone or combined surgery)
- LVEF <30 %, or 30 % ≤ LVEF ≤ 40 %
- Presence of a preoperative treatment with beta-blockers.

The heterogeneity of the treatment effect will be evaluated by an interaction test and presented using a forest plot [16].

## Discussion

### Rationale for levosimendan in cardiac surgical patients with poor LV function

Levosimendan was introduced a few years ago and was initially approved in some countries for the management of acutely decompensated heart failure following the results of two multicenter randomized controlled trials (LIDO and SURVIVE) [17, 18]. Preliminary data on the beneficial effects of levosimendan in cardiac surgical patients has been reported in several studies [7–9, 15, 19–24]. However, this data was obtained from trials conducted at single centers [8, 19–24], on small cohorts (<100 patients) [8, 9, 20–22, 24], without randomization [24], or according to a single-blinded protocol [7, 9, 15, 22–24]. Therefore, the level of evidence supporting the beneficial effect of levosimendan is not very strong.

In a small randomized study, patients with a preoperative LVEF ≤30 % treated with levosimendan received a smaller amount of dobutamine in comparison to those treated with milrinone and had a lower mortality rate after surgery [9]. In another prospective, randomized, double-blind study comparing levosimendan to a placebo in 60 patients with a preoperative LVEF <50 % undergoing CABG, levosimendan was found to enhance primary weaning from cardiopulmonary bypass in comparison to the placebo [8]. Levin et al. compared preoperative levosimendan versus placebo in 252 CABG patients with a LVEF <25 % and found that levosimendan reduced mortality (3.9 vs 12.8 %; *p* < 0.05), the incidence of LCOS (7.1 vs 20.8 %; *p* < 0.05) and of complicated weaning from cardiac pulmonary bypass (2.4 vs 9.6 %; *p* < 0.05). Compared to the placebo group, the levosimendan-treated patients also required less inotropes (7.9 vs 58.4 %; *p* < 0.05), vasopressors (14.2 vs 45.6 %; *p* < 0.05), and intra-aortic balloon pumping (6.3 vs 30.4 %; *p* < 0.05) [7]. A recent meta-analysis of perioperative use of levosimendan confirmed that the efficacy of levosimendan seems to be more pronounced in patients with preoperative left ventricular dysfunction [12].

### Study population

In addition to CABG surgery, we deliberately choose to enrol patients undergoing combined surgery despite the fact that they might introduce some variability in our population. Indeed, a patient with aortic stenosis and poor left ventricular function associated with coronary artery disease is likely to have a myocardial dysfunction related to the long lasting pressure overload in addition to myocardial ischemia. Ischemic mitral insufficiency combines chronic volume overload and ischemia, and the correction of mitral regurgitation (valve repair or replacement) will adversely affect left ventricular afterload. Furthermore, these combined procedures are inherently associated with longer aortic cross-clamping times which, in turn, may increase the probability of a LCOS, as reflected by the higher mortality rate in this population [25]. We have no data to determine whether levosimendan is more likely to have beneficial effects in one subgroup or another. The cardio protective effect of mitochondrial K_ATP_ channel opening might be more effective in patients with pure ischemic cardiomyopathy. But the vasodilatation resulting from vascular smooth muscle K_ATP_ channel opening and the inotropic effect due to calcium sensitizing of the myofilaments are expected to be beneficial regardless of the mechanism of LV failure. The respective importance of each pharmacologic effect of levosimendan is not clearly identified. The minimization process used for randomization takes into account the fact that CABG is isolated or combined with a valve repair and should help in addressing this question of the efficacy of the drug in relation to the underlying type of pathology. Another reason for being “less selective” is to facilitate recruitment and to maximize the number of eligible patients over a relatively short period of time. This is an important issue as levosimendan must be used within 24 months after production. Broadening of inclusion criteria is also likely to generate outcome data more reliably representative of current practice in the “real world”.

### Pharmacokinetics of levosimendan

Levosimendan has a linear pharmacokinetic profile with an elimination half-life of about one hour. Maximum plasma levosimendan concentration is reached 4.4 h after an infusion of 0.2 μg/kg/min over 6 h. Maximum plasma concentration of its main metabolite (OR-1896) is reached 2–5 days after an infusion of 0.2 μg/kg/min over 24 h. Since OR-1896 has a similar haemodynamic profile to levosimendan itself, this explains the prolonged effect of the drug. Levosimendan is extensively metabolized by the liver and inactive metabolites are excreted in the urine and the faeces within 24 h [26]. For patients with renal impairment, defined as a glomerular filtration rate between 30 and 60 mL/min/1.73 m^2^, the dose of levosimendan does not need to be reduced and the drug even seems to improve kidney function in patients with acute decompensated heart failure [27]. Interestingly, the beneficial haemodynamic effects of levosimendan, unlike those of dobutamine, are not attenuated by the concomitant use of β-blockers [18].

### Side effects of levosimendan and safety issues

According to the clinical data of REVIVE II and SURVIVE trials, moderate hypotension (mean reduction in systolic blood pressure: −4 mmHg, and diastolic blood pressure: − 6 mmHg) was the most frequent adverse event observed after a bolus infusion of levosimendan (12 μg/kg) and lasted for 12 h [17, 28]. In the SURVIVE trial, the occurrence of hypotension did not differ between the levosimendan and dobutamine groups. However, based on this finding we will not administer any bolus of levosimendan in our patients in order to reduce the risk of intra-operative hypotension. Previous authors using a continuous infusion of levosimendan without a loading dose observed beneficial effects without hypotension [9, 22, 24]. Moreover, 2012 European Society of Cardiology guidelines for the diagnosis and treatment of acute and chronic heart failure recommended the use of levosimendan without a loading dose for hypotensive patients [29].

Levosimendan administration has also been associated with a higher incidence of atrial fibrillation in comparison to a placebo (9 vs 2 %) [28] or dobutamine (9 vs 6 %) [17] in trials involving patients with left ventricular failure. In contrast, meta-analysis of studies involving surgical patients suggested a reduced incidence of atrial fibrillation in the group of patients treated with levosimendan [30]. We will collect information on the incidence of atrial fibrillation in our patients to try to provide new insight regarding these discrepant results. Atrial fibrillation detection will rely on continuous electrocardiography monitoring while in ICU, and on clinically patent arrhythmia episodes leading to 12-lead electrocardiography recording while in the ward. Information regarding other arrhythmias (ventricular tachycardia, ventricular fibrillation, torsade de pointe) will also be collected to assess the safety profile of levosimendan in the perioperative period.

Perioperative ischemia is a major concern after CABG surgery. The safety profile of levosimendan regarding the myocardial oxygen balance seems favourable in comparison to dobutamine. Previous studies involving CABG patients suggested that postoperative ischemia was less frequent in levosimendan-treated patients with respect to a placebo [7, 19]. Peak postoperative troponin I (between Day 1 and Day 2), measured by highly-sensitive troponin assay will be monitored at each center to assess myocardial damage [31].

## Conclusion

This trial is intended to answer an important question: is there a role for levosimendan as a pre-treatment for patients with poor left ventricular function (LVEF ≤40 %) undergoing CABG (alone or combined with valve surgery) under cardiopulmonary bypass? The primary objective of the study is to quantify the occurrence of simple clinical markers of low cardiac output syndrome in the levosimendan and placebo groups. If levosimendan proves superior to a placebo, this will result in a change of management for high risk cardiac surgical patients, which is clinically relevant in view of the increased prevalence of this patient cohort.

## Abbreviations

CABG, coronary artery bypass grafting; CONSORT, Consolidated Standards of Reporting Trials; CRF, case report forms; IABP, intra-aortic balloon pump; ICU, intensive care unit; LCOS, low cardiac output syndrome; LVEF, left ventricular ejection fraction; MAP, mean arterial pressure; RRT, renal replacement therapy
